# Metagenomic and culturomic analysis of gut microbiota dysbiosis during *Clostridium difficile* infection

**DOI:** 10.1038/s41598-019-49189-8

**Published:** 2019-09-05

**Authors:** Sophie Amrane, Marie Hocquart, Pamela Afouda, Edmond Kuete, Thi-Phuong-Thao Pham, Niokhor Dione, Issa Isaac Ngom, Camille Valles, Dipankar Bachar, Didier Raoult, Jean Christophe Lagier

**Affiliations:** Aix Marseille Univ, IRD, MEPHI, IHU-Méditerranée Infection, Marseille, France

**Keywords:** High-throughput screening, Bacteriology, Diarrhoea

## Abstract

Recently, cocktail of bacteria were proposed in order to treat *Clostridium difficile* infection (CDI), but these bacteriotherapies were selected more by chance than experimentation. We propose to comprehensively explore the gut microbiota of patients with CDI compared to healthy donors in order to propose a consortium of bacteria for treating *C*. *difficile*. We compared stool samples composition from 11 CDI patients and 8 healthy donors using two techniques: metagenomics, 16S V3-V4 region amplification and sequencing and culturomics, high throughout culture using six culture conditions and MALDI-TOF identification. By culturomics, we detected 170 different species in the CDI group and 275 in the control group. *Bacteroidetes* were significantly underrepresented in the CDI group (p = 0.007). By metagenomics, 452 different operational taxonomic units assigned to the species level were detected in the CDI group compared to 522 in the control group. By these two techniques, we selected 37 bacteria only found in control group in more than 75% of the samples and/or with high relative abundance, 10 of which have already been tested in published bacteriotherapies against CDI, and 3 of which (*Bifidobacterium adolescentis*, *Bifidobacterium longum* and *Bacteroides ovatus)* have been detected by these two techniques. This controlled number of bacteria could be administrated orally in a non-invasive way in order to treat CDI.

## Introduction

*Clostridium difficile* is responsible for human diseases ranging from mild diarrhea to pseudomembranous colitis^1^. *C*. *difficile* was responsible for almost 30,000 deaths in the USA in 2011^2^, illustrating the high morbimortality of the disease and an increase in the number of cases. Gut dysbiosis is the triggering factor of *C*. *difficile* infection (CDI)^3,4^. One of the current treatments, fecal microbiota transplantation (FMT), is based on the restoration of a healthy microbiota^5^. FMT demonstrated its effectiveness in a randomized study^5^ with 81% of recovery after treatment. FMT is currently recommended for recurrent CDI^6^. FMT has also demonstrated its superiority compared with antibiotics as first-line treatment for severe CDI^7^.

Nevertheless, FMT using whole stool samples presents some limitations. For instance, despite an important pathogen screening among donors^6^, pathogen transmission through entire stool donations remains possible^8,9^. Oral administration by capsules has been proposed^10^ but usual methods of administration (nasogastric tube, colonoscopy…) remain invasive^11^. Rare but serious adverse events correlated to these routes of administration have been reported: aspirating pneumonia, rectal perforation^11^. Although there is no formal evidence, some gut bacteria have been associated to colorectal cancer^12^ or obesity^13^. An unexplained gain of 8.5 points of BMI following FMT has been reported^14^. It is therefore desirable to know exactly which bacteria are transferred to the patient.

In order to overcome these weaknesses, some authors proposed bacteriotherapies as treatment against CDI (see Table 1). Petrof *et al*. successfully administrated to two patients with recurrent CDI a cocktail of 33 bacteria^15^ selected after culture of a stool from a healthy subject in strict anaerobic conditions. Tvede *et al*. selected twelve bacteria; some of them were shown to inhibit *C*. *difficile* growth *in vitro*. The mixture was administrated to 55 patients with recurrent CDI, with 63% of success^16^. SER-109, an experimental treatment, containing about fifty spore-forming bacteria, was administrated for prevention of recurrent CDI with 86% of success^17^. In order to promote sporulation and kill potential pathogens, the authors chose to treat stool samples from healthy donors with ethanol^17^. Currently, there is no commercialized bacteriotherapy for CDI treatment. In addition, in all the bacteriotherapies offered, the bacteria used are selected more by chance than by empirical experimentation^18^.

**Table 1:** Bacteria used in other bacteriotherapy studies^15–17^ against CDI.

Petrof *et al*.	Tvede *et al*.	Khanna et al.
Actinobacteria:	Bacteroidetes	Firmicutes
*Bifidobacterium adolescentis (2)*	*Bacteroides ovatus*	*Butyricicoccus sp*.
*Bifidobacterium longum (2)*	*Bacteroides thetaiotaomicron*	*Clostridium sp*.
*Collinsella aerofaciens*	*Bacteroides vulgatus*	*Hungatella sp*.
		*Flavonifractor sp*.
Bacteroidetes	Firmicutes	*Unclassified Clostridiales genera*
*Bacteroides ovatus*	*Clostridium bifermentans*	*Coprobacillus sp*.
*Parabacteroides distasonis*	*Clostridium innocuum*	*Erysipelatoclostridium sp*.
	*Clostridium ramosum*	*Holdemanella sp*.
Firmicutes	*Enterococcus faecalis*	*Solobacterium sp*.
*Acidaminococcus intestinalis*	*LactobacilIus acidophilus*	*Turicibacter sp*.
*Clostridium cocleatum (91,92%)*	*LactobacilIus rhamnosus*	*Anaerofustis sp*.
*Eubacterium desmolans (94,9%)*	*Lactobacillus sp*	*Eubacterium sp*.
*Eubacterium eligens (98,15%)*		*Anaerosporobacter sp*.
*Eubacterium limosum (97,05%)*	Proteobacteria	*Anaerostipes sp*.
*Eubacterium rectale (4)*	*Escherichia coli* (2)	*Blautia sp*.
*Eubacterium ventriosum*		*Coprococcus sp*.
*Blautia producta (96,43%)*		*Dorea sp*.
*Dorea longicatena (2)*		*Lachnobacterium sp*.
*Lachnospira pectinoshiza (95,22%)*		*Lachnoclostridium sp*.
*Roseburia faecalis*		*Lachnospira sp*.
*Roseburia intestinalis*		*Roseburia sp*.
*Lactobacillus casei*		*Tyzzerella sp*.
*Lactobacillus casei/paracasei*		*Oscillibacter sp*.
*Faecalibacterium prausnitzii*		*Intestinibacter sp*.
*Ruminococcus obeum (94,69%)*		*Peptoclostridium sp*.
*Ruminococcus obeum (94,89%)*		*Terrisporobacter sp*.
*Ruminococcus torques (2)*		*Acetivibrio sp*.
*Streptococcus mitis*		*Anaerotruncus sp*.
		*Faecalibacterium sp*.
Proteobacteria		*Gemmiger sp*.
*Escherichia coli*		*Oscillospira sp*.
*Raoultella sp*.		*Ruminiclostridium sp*.
		*Ruminococcus sp*.
		*Sporobacter sp*.
		*Subdoligranulum sp*.

Metagenomics is the gold standard method to study gut microbiota^19,20^, but a large part of the detected bacteria has never been cultivated^21^. The culturomic approach^22^ allowed an increase of known gut bacteria. Using this method, 766 bacteria were added to the human gut repertoire and 247 of them where cultivated for the first time during these experiments^23^. Recently, through culturomic analysis, *Clostridium butyricum* has been suggested as the agent responsible for necrotizing enterocolitis in infants^24^.

In this study, we propose to comprehensively explore, by metagenomics and culturomics, the gut microbiota of patients with CDI compared to healthy donors. The aim of this work is to highlight a consortium of bacteria capable of fighting against *C*. *difficile*.

## Results

### Differences in gut microbiota composition between CDI and controls

#### Culturomic results

We analyzed eleven stool samples from patients with CDI, eight of which underwent ethanol preincubation. Clinical characteristics of the patients are summarized in Supplementary Table S2. Among the patients with available clinical data, all underwent antibiotic treatment before their first CDI. The median age was 65 and seven were in a relapse situation. We also analyzed eight stool samples from controls, including six with and without ethanol preincubation. All bacteria detected in this culturomic study are summarized in the Supplementary Table S3.

#### Comparison between CDI and control group

**Figure 1:**
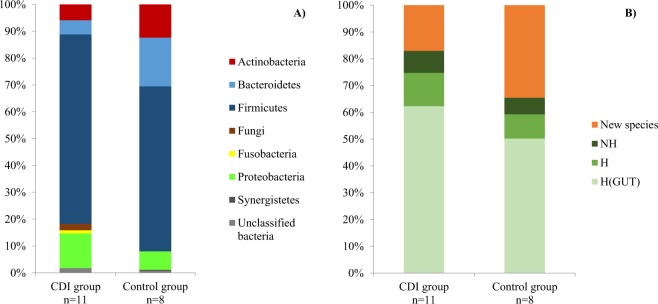
(**A**) Phyla repartition between bacteria detected in culturomic analysis in CDI and control group. (**B**) Proportion of bacteria in both group identified by culturomics and already known in human gut microbiota composition (H(GUT)), already known in human other site (H), first isolated in human (NH) and new species discovered by culturomic studies.

We analyzed eleven stool samples from patients with CDI (including eight with ethanol preincubation) and eight stool samples from healthy controls (including six with ethanol preincubation). In the CDI group, we obtained 170 different species with a mean of 40 species per sample. In the control group, we obtained 275 different species with a mean of 86 bacteria per sample. Phyla repartition in both groups is presented in Fig. 1A. We observed a significant depletion of *Bacteroidetes* in the CDI group (n = 9/170, 5.3%) compared to the control group (n = 50/275, 18.2%) with p = 0.007 (Fisher exact test). Depletion of *Actinobacteria* (5.9% versus 12.4%) and increase of *Proteobacteria* (12.9% versus 6.9%) in the CDI group were not significant (respectively p = 0.2 and p = 0.24). In both groups, most of the bacteria detected were already known and cultivated from the human gut, but a large part was also represented by bacteria newly described during previous and current culturomic studies (Fig. 1B). This part was more abundant in the control group (n = 95/275, 34.6%) than in the CDI group (n = 29/170, 17%). Only 80 bacterial species were common to both groups. Most of them (n = 62/80, 78%) belonged to *Firmicutes* (Supplementary Data Table S3), which is the most represented phyla in gut microbiota.

#### Metagenomic results

Sample description and microbial diversity: We analyzed by metagenomic eleven stool samples from patients with CDI and eight stool samples from healthy controls. We obtained a total of 1,964,703 reads distributed between 2,318 operational taxonomic units (OTUs) taxonomically assigned with more than 80% of similarity with a domain of bacteria or archea. 77.7% (1,525,987) of these reads could be related to OTUs taxonomically assigned at the species level with more than 98% similarity. In the CDI group, we found a mean of 70,218 reads per sample. 88.4% of these reads were assigned to a known species with 452 different species detected in this group (with a mean of 88 species per sample, min 37-max 193). In the control group, we found a mean of 148,997 reads per sample. 70.7% of these reads were assigned to a known species with 522 different species detected in this group (with a mean of 161 species per sample, min 129-max 197).

**Figure 2:**
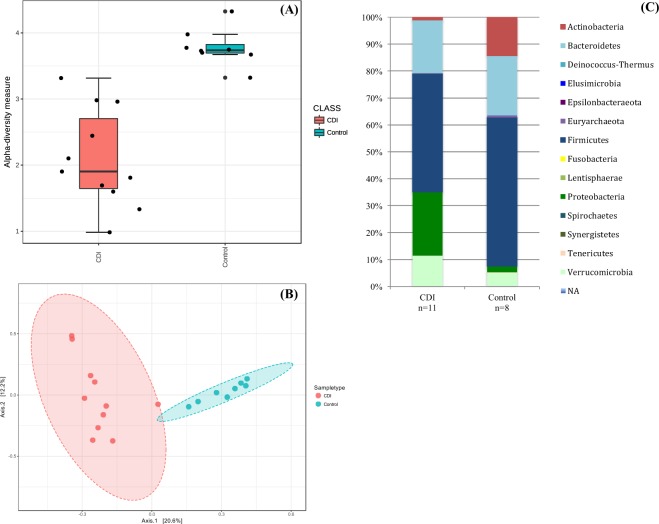
(**A**) Shannon index, p = 9.0334e-06 (t-test), (**B**) PCoA, (**C**) Phyla repartition between CDI group and control group in metagenomic analysis^49^.

We calculated the Shannon index (marker of intra-individual diversity) with overall reads. Diversity was significantly higher in the control group (p = 0.000009, t-test) (Fig. 2A).

Microbial community structure: Principal coordinate analysis (PCoA) was performed with all OTUs (Fig. 2B). Microbiota composition was different for the two groups. Within each group, the composition of most samples was closer to that of other members of the group rather than to the members of the other group.

Among the 2,318 different OTUs, 728 were detected in both groups, 1,049 were detected only in the control group and 541 were detected only in the CDI group (Supplementary Data Table S4). *Clostridium difficile* was only detected in the CDI group, in 8/11 (72.7%) samples.

Species repartition between CDI and control group: Phyla repartition between CDI and control group was different (Fig. 2C). *Proteobacteria* were more common in the CDI group (23.2%) compared to the control group (2.2%). *Firmicutes* and *Actinobacteria* were less common in the CDI group (respectively 33.5% and 1.4%) compared to the control group (respectively 55.2% and 14.3%).

### Proposition of bacteria associated with a healthy microbiota

#### Culturomics

**Figure 3:**
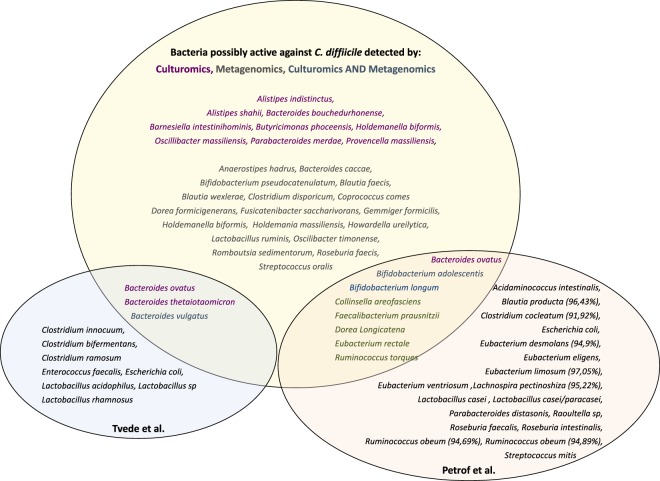
Bacteria associated to healthy microbiota, found by culturomics, metagenomics, culturomics and metagenomics, and compared to other published bacteriotherapies.

We compared all bacteria obtained in the CDI group and in the control group (with and without ethanol preincubation). Bacteria found only in the control group may have a role against *C*. *difficile*. Among those bacteria, *Bacteroides ovatus*, *Bacteroides vulgatus* and *Oscillibacter massiliensis* were found in all the control samples. Fourteen bacteria were found in at least 75% of the control samples (Fig. 3). Among these 14 bacteria, 9 (64.3%) belonged to the phylum *Bacteroidetes*, 3 (21.4%) to the phylum *Firmicutes*, 9 (64.3%) had already been cultivated from the human gut and 5 (35.7%) had been discovered by previous culturomic studies. Moreover, five bacteria (Fig. 3) in this list had already been used by other authors in bacteriotherapies^15,16^.

#### Metagenomics

We performed a linear discriminant analysis (LDA) for comparison between CDI and control group (Supplementary Fig. S1). Among the 1,049 OTUs present only in the control group, 20 were relatively abundant with values superior to 0.1% and were present in at least 75% of the control samples (Supplementary Fig. S2), they may also play a role against *C*. *difficile*. By regrouping these data, we found 35 different OTUs associated with a healthy microbiota. Eight bacteria (Fig. 3) of this group had already been used in bacteriotherapies^15,16^.

#### Comparison between metagenomic and culturomic results

Among the 35 bacteria detected by metagenomics and associated with a healthy microbiota, 26 have been identified up to the species level and are currently cultivable (Fig. 3). By culturomics, we detected 14 bacteria associated with a healthy microbiota (Fig. 3). Only three bacteria with a potential role against *C*. *difficile* were detected by both culturomics and metagenomics, namely *Bifidobacterium adolescentis*, *Bifidobacterium longum* and *Bacteroides ovatus*. Thus, 37 cultivable bacteria were selected by these two technics, ten of them have already been used as bacteriotherapies against *C*. *difficile* (Fig. 3)^15,16^.

## Discussion

Dysbiosis during CDI has already been analyzed by metagenomics several times^25^. This technique is the gold standard method for gut microbiota studies^19,20^. The culturomic approach has been developed since 2012 in our laboratory^23,26^. To broaden our field of vision on gut microbiota modification during CDI, we have chosen to apply these two different methods to the same stool samples. Both analyses showed a disrupt microbiota during CDI with decreased richness and diversity. This results were concordant with former published study on microbiota composition during CDI infection^25^. *Firmicutes* are implicated in butyrate and short chain fatty acid production, this molecules play a role in gut homeostasis and inhibition of *C*. *difficile* germination^25,27^. *Bacteroidetes* are implicated in carbohydrates digestion, producing substrates for colonocytes^25^. Depletion of these two major phyla of gut microbiota was detected in our analyses among CDI group. To the best of our knowledge, this double approach had never been described for dysbiosis caused by *C*. *difficile*. Our experience in culturomics^23^ enabled us to select the most profitable growing conditions. In order to reduce the extraction bias of the metagenomic technique^28^, we used two different protocols for DNA extraction. We obtained metagenomic results of good quality, more than 75% of the detected OTUs were assigned to a known species with more than 98% similarity. Our data were not compared to other available dataset of gut microbiota analysis during CDI. Metagenomic data comparison is complex because of high heterogeneity of the used technics and various sequencing depth used^29^.

In this study, culturomics allowed the detection of a total of 356 different bacteria, 112 (31.5%) of which were new species, detected for the first time by current or previous culturomics studies. This approach expands our knowledge of unknown parts of gut microbiota. Only 88 bacteria were detected by both culturomics and metagenomics, representing 24.7% of the bacteria in the culturomic approach and 11.9% of the OTUs assigned to a known species in the metagenomic approach. This low similarity percentage between the two approaches demonstrates the value of using complementary methods for gut microbiota description^26^. Among the 37 detected bacteria with a potential role against *C*. *difficile*, only three (*Bacteroides vulgatus*, *Bifidobacterium adolescentis*, *Bifidobacterium longum*) were detected by the two methods.

By comparing the bacteria evidenced by our work with those from other bacteriotherapies proposed against *C*. *difficile*, we found bacteria in common. Five bacteria detected by our culturomic study have already been tested in bacteriotherapies. *Bacteroides ovatus*, *Bacteroides thetaiotaomicron* and *Bacteroides vulgatus* have been used by Tvede *et al*.^16^. *Bacteroides ovatus*, *Bifidobacterium adolescentis*, *Bifidobacterium longum*, have been used by Petrof *et al*.^15^. In the same way, eight bacteria detected by our metagenomic study have been tested by Petrof *et al*.^15^: *Bifidobacterium adolescentis*, *Faecalibacterium prausnitzii*, *Bifidobacterium longum*, *Collinsella aerofaciens*, *Eubacterium rectale*, *Ruminococcus torques* and *Dorea longicatena* and Tvede *et al*.^16^: *Bacteroides vulgatus*.

Among the panel of bacteria proposed against *C*. *difficile* with an available taxonomic classification, *Bacteroidaceae*, *Bifidobacteriaceae* and *Lachnospiraceae* are the three most represented families. These families are associated with resistance against *C*. *difficile*^30–33^. Two of the three bacteria detected by metagenomics and culturomics belong to the *Bifidobacterium* genus, which is associated with a healthy microbiota^34,35^. *Bifidobacterium* species are largely used as probiotics^36^. *Bifidobacterium bifidum* is associated with prevention of the first episode of CDI^37^.

This original work proposed the first comprehensive analysis of gut microbiota modification during CDI using culturomics and metagenomics. Using these two methods, we detected 37 cultivable bacteria with a potential role against *C*. *difficile*. More than one super probiotic, a consortium of bacteria able to restore normal gut flora seems interesting. Indeed all stool preparation for FMT are not as efficient, Ruminococcaceae and Lachnospiraceae seems to play a major role in gut microbiota restoration^38^. Not only bacteria but also environment and interactive molecules produced by them are effective for restoration of gut homeostasis^39^.

Further explorations are needed before probiotic utilization. Gut microbiota analysis could be performed before and after FMT for CDI patient. The consortium of protective bacteria might be tested on an animal model before human administration. If confirmed, this controlled number of bacteria could be administrated orally in a non-invasive way^10^.

## Methods

### Patients and samples

Our study involved 19 stool samples divided in two groups: one CDI group and one control group. CDI was defined as diarrhea along with a positive detection of *C*. *difficile* B toxin by real-time PCR (RT-PCR) in the stool samples. We used the RT-PCR Xpert® *C*. *difficile* (Cepheid, Sunnyvale, USA) targeting toxin genes tcdB and cdt and detecting 027 genotype by tcdC gene deletion at nt 117. For each positive sample, we searched 078 genotype by tcdC gene amplification and sequencing to detect a 39 base pair deletion and a mutation in position 184^40^. In our laboratory, all the *C*. *difficile* positive stool samples are frozen at −80 °C for conservation. The ethics committee of the Institut Hospitalo-Universitaire Mediterranée Infection approved the use of this data under the agreement number 2016-011. Informed consent was obtained from all patients.

For the CDI group, we selected three stool samples: one of 027 genotype, one of 078 genotype and one of neither 027, nor 078 genotype. Nine additional *C*. *difficile*-positive stool samples were selected over time in our point-of-care laboratory in order to work with fresh samples. All the clinical data were collected from the digitized patient record of the IHU Mediterranée Infection (Axigate software).

As for the control group, we used stool samples from eight voluntary healthy adults that were stool donor for FMT. They were unrelated to CDI patient, not obese and aged between 18 and 45 years old. Their stools were tested negative for *C*. *difficile* by RT-PCR. In our center, between April and December 2017, their stools were used for 32 FMT for 28 different patients with CDI. At 6 month, follow-up was available for 23 patients. Among them, 65.2% were cured by one FMT, 34.8% relapsed after FMT.

### General strategy

We proposed to analyze stool samples from patients with CDI and healthy donors with two different approaches: metagenomics and culturomics. For each analytical condition, we proposed to evaluate the differences in the composition of the gut microbiota between the two groups. Using the culturomic approach, in order to compare our work with bacteriotherapies against *C*. *difficile*^15–17^, we worked under aerobic and anaerobic conditions and promoted bacterial sporulation with ethanol. With this multiple approach of gut microbiota composition, we selected for each analysis condition the gut bacteria associated with resistance against *C*. *difficile*. The protocol was carried out in accordance with relevant guidelines and regulations and was approved by our ethic committee.

### Culturomics analysis

We performed high throughput culture^22^ using six culture conditions with preincubation in blood culture bottle and culture on blood agar (Supplements Table 1). As already described^22,23^, for each sample, one gram of stool was inoculated in a blood bottle with adjunction of rumen, sheep blood or both rumen and sheep blood. At regular intervals, a ten-fold serial dilution of the culture medium was performed and inoculated on Columbia agar with 5% sheep blood (Bio Mérieux, Marcy-L’Etoile, France). Each pure colony obtained after subculture was identified using Matrix Assisted Laser Desorption/Ionization Time of Flight Mass Spectrometry (MALDI-TOF MS)^41^. If, despite the good spectra quality, the bacteria has not been identified, a 16S rDNA gene sequencing of the bacteria has been performed. DNA was extracted using EZ1 DNA Tissue Kit (Qiagen, Venlo, Netherland) on EZ1 automat (Qiagen). 16 S amplification and sequencing were performed as described elsewhere^42^. A sequence homology lower than 98.65% between the studied bacterial strain and strains described in the literature defined potential new species^43^.

In order to promote bacterial sporulation, we used a protocol of stool pretreatment with ethanol, following the indications by Browne *et al*.^44^. Stools were incubated with 70% ethanol v/v with DPBS (Dulbecco’s Phoshate Buffered Saline, Gibco®, Thermo Fisher Scientific) during four hours. After the incubating period, the supernatant was removed and the remaining stools were rinsed with DPBS and inoculated into blood bottles to implement the culturomic protocol, as previously described. This procedure was performed for eight CDI samples and six control samples.

### Metagenomics analysis

Stool samples were analyzed with metagenomics focused on the V3-V4 of the 16S DNA gene thanks to the MiSeq® technology (Illumina, San Diego, USA). Two protocols, number 1 and 5, were used for total DNA extraction as previously described^28^. We used the same protocol for sequencing and bioinformatic analysis, as previously described^23^.

Obtained OTUs were blasted^45^ against the database of SILVA^46^ (release 128) and the matches with identity ≥80% and 100% coverage were extracted from the reference database. The taxonomy was assigned by applying majority voting^47,48^, considering species level at identity ≥98%, genus level ≥97% identity, family ≥95% identity and so on. Each unassigned OTU at the species level was named with a number and its similarity percentage with the closest species was detected. OTUs were aligned against our homemade database, made of all the bacterial sequences newly detected from the previous culturomic studies.

### Statistical analysis

Statistical analyses were performed with Marker Data Profiling on MicrobiomeAnalyst software^49^. Shannon index was calculated using t-test and PCoA was calculated using Bray Curtis index between samples. LDA was performed on the Huttenhower Lab website^50^ with LEfse tool^51^. We used 0.05 alpha value for the factorial Kruskal-Wallis test among classes and for the pairwise Wilcoxon test between subclasses.

### Ethical Approval

This study was approved by ≪ the ethics comity of the Institut Hospitalo-Universitaire Mediterranée Infection ≫ under number 2016-011.

## Supplementary information

Supplementary data

Dataset 1

## Data Availability

The dataset used is available in supplementary data.

## References

[1] Debast SB, Bauer MP, Kuijper EJ (2014). European Society of Clinical Microbiology and Infectious Diseases: Update of the Treatment Guidance Document for Clostridium difficile Infection. Clin. Microbiol. Infect..

[2] Lessa FC (2015). Burden of Clostridium difficile Infection in the United States. N. Engl. J. Med..

[3] Vincent C (2013). Reductions in intestinal Clostridiales precede the development of nosocomial Clostridium difficile infection. Microbiome.

[4] Seekatz AM, Rao K, Santhosh K, Young VB (2016). Dynamics of the fecal microbiome in patients with recurrent and nonrecurrent Clostridium difficile infection. Genome Med..

[5] van Nood E (2013). Duodenal Infusion of Donor Feces for Recurrent Clostridium difficile. N. Engl. J. Med..

[6] Cammarota G (2017). European consensus conference on faecal microbiota transplantation in clinical practice. Gut.

[7] Hocquart, M. *et al*. Early Faecal Microbiota Transplantation Improves Survival in Severe Clostridium difficile Infections. *Clin*. *Infect*. *Dis*. (2017). 10.1093/cid/cix762 29020328

[8] Martin J, Wilcox M (2016). New and emerging therapies for Clostridium difficile infection. Curr. Opin. Infect. Dis..

[9] Schwartz M, Gluck M, Koon S (2013). Norovirus Gastroenteritis After Fecal Microbiota Transplantation for Treatment of Clostridium difficile Infection Despite Asymptomatic Donors and Lack of Sick Contacts. Am. J. Gastroenterol..

[10] Staley, C. *et al*. Successful Resolution of Recurrent Clostridium difficile Infection using Freeze-Dried, Encapsulated Fecal Microbiota; Pragmatic Cohort Study. *Am*. *J*. *Gastroenterol* (2017). 10.1038/ajg.2017.6 PMC5552199 28195180

[11] Baxter M, Colville A (2016). Adverse events in faecal microbiota transplant: a review of the literature. J. Hosp. Infect..

[12] Viljoen KS, Dakshinamurthy A, Goldberg P, Blackburn JM (2015). Quantitative Profiling of Colorectal Cancer-Associated Bacteria Reveals Associations between Fusobacterium spp., Enterotoxigenic Bacteroides fragilis (ETBF) and Clinicopathological Features of Colorectal Cancer. PLoS One.

[13] Armougom F, Henry M, Vialettes B, Raccah D, Raoult D (2009). Monitoring Bacterial Community of Human Gut Microbiota Reveals an Increase in Lactobacillus in Obese Patients and Methanogens in Anorexic Patients. PLoS One.

[14] Alang N, Kelly CR (2015). Weight Gain After Fecal Microbiota Transplantation. Open Forum Infect. Dis..

[15] Petrof EO (2013). Stool substitute transplant therapy for the eradication of Clostridium difficile infection: ‘RePOOPulating’ the gut. Microbiome.

[16] Tvede M, Tinggaard M, Helms M (2015). Rectal bacteriotherapy for recurrent Clostridium difficile-associated diarrhoea: results from a case series of 55 patients in Denmark 2000–2012. Clin. Microbiol. Infect..

[17] Khanna S (2016). A Novel Microbiome Therapeutic Increases Gut Microbial Diversity and Prevents Recurrent Clostridium difficile Infection. J. Infect. Dis..

[18] Lagier J-C, Cadoret F, Raoult D (2017). Critical Microbiological View of SER-109. J. Infect. Dis..

[19] Turnbaugh PJ (2007). The human microbiome project. Nature.

[20] Dhakan, D. B. *et al*. The unique composition of Indian gut microbiome, gene catalogue, and associated fecal metabolome deciphered using multi-omics approaches. *Gigascience* **8** (2019). 10.1093/gigascience/giz004 PMC6394208 30698687

[21] Eckburg PB (2005). Diversity of the Human Intestinal Microbial Flora. Science (80-.)..

[22] Lagier J (2015). The Rebirth of Culture in Microbiology through the Example of Culturomics To Study Human Gut Microbiota. Clin. Microbiol. Rev..

[23] Lagier J-C (2016). Culture of previously uncultured members of the human gut microbiota by culturomics. Nat. Microbiol..

[24] Cassir N (2015). Clostridium butyricum Strains and Dysbiosis Linked to Necrotizing Enterocolitis in Preterm Neonates. Clin. Infect. Dis..

[25] Zhang L (2015). Insight into alteration of gut microbiota in Clostridium difficile infection and asymptomatic C. difficile colonization. Anaerobe.

[26] Lagier J (2012). Microbial culturomics: paradigm shift in the human gut microbiome study. Clin. Microbiol. Infect..

[27] Abt MC, McKenney PT, Pamer EG (2016). Clostridium difficile colitis: pathogenesis and host defence. Nat. Rev. Microbiol..

[28] Angelakis E (2016). Glycans affect DNA extraction and induce substantial differences in gut metagenomic studies. Sci. Rep..

[29] Lagier J-C, Dubourg G, Amrane S, Raoult D (2017). Koch Postulate: Why Should we Grow Bacteria?. Arch. Med. Res..

[30] Antharam VC (2013). Intestinal Dysbiosis and Depletion of Butyrogenic Bacteria in Clostridium difficile Infection and Nosocomial Diarrhea. J. Clin. Microbiol..

[31] Lee YJ (2017). Protective Factors in the Intestinal Microbiome Against Clostridium difficile Infection in Recipients of Allogeneic Hematopoietic Stem Cell Transplantation. J. Infect. Dis..

[32] Schubert AM (2014). Microbiome Data Distinguish Patients with Clostridium difficile Infection and Non-C. difficile-Associated Diarrhea from Healthy Controls. MBio.

[33] Milani C (2016). Gut microbiota composition and Clostridium difficile infection in hospitalized elderly individuals: a metagenomic study. Sci. Rep..

[34] Hidalgo-Cantabrana, C. *et al*. Bifidobacteria and Their Health-Promoting Effects. *Microbiol*. *Spectr*. **5** (2017). 10.1128/microbiolspec.bad-0010-2016 PMC11687494 28643627

[35] McFarland LV (2015). Probiotics for the Primary and Secondary Prevention of C. difficile Infections: A Meta-analysis and Systematic Review. Antibiot. (Basel, Switzerland).

[36] Hill C (2014). Expert consensus document: The International Scientific Association for Probiotics and Prebiotics consensus statement on the scope and appropriate use of the term probiotic. Nat. Rev. Gastroenterol. Hepatol..

[37] Milani C (2015). Bifidobacteria exhibit social behavior through carbohydrate resource sharing in the gut. Sci. Rep..

[38] Wilson BC, Vatanen T, Cutfield WS, O’Sullivan JM (2019). The Super-Donor Phenomenon in Fecal Microbiota Transplantation. Front. Cell. Infect. Microbiol..

[39] Ott SJ (2017). Efficacy of Sterile Fecal Filtrate Transfer for Treating Patients With Clostridium difficile Infection. Gastroenterology.

[40] Cassir, N. *et al*. Emergence of Clostridium difficile tcdC variant 078 in Marseille, France. *Eur*. *J*. *Clin*. *Microbiol*. *Infect*. *Dis*. (2017). 10.1007/s10096-017-3022-8 28573471

[41] Seng P (2009). Ongoing Revolution in Bacteriology: Routine Identification of Bacteria by Matrix‐Assisted Laser Desorption Ionization Time‐of‐Flight Mass Spectrometry. Clin. Infect. Dis..

[42] Dubourg G (2014). Culturomics and pyrosequencing evidence of the reduction in gut microbiota diversity in patients with broad-spectrum antibiotics. Int. J. Antimicrob. Agents.

[43] Kim M, Oh H-S, Park S-C, Chun J (2014). Towards a taxonomic coherence between average nucleotide identity and 16S rRNA gene sequence similarity for species demarcation of prokaryotes. Int. J. Syst. Evol. Microbiol..

[44] Browne HP (2016). Culturing of ‘unculturable’ human microbiota reveals novel taxa and extensive sporulation. Nature.

[45] Altschul SF, Gish W, Miller W, Myers EW, Lipman DJ (1990). Basic local alignment search tool. J. Mol. Biol..

[46] Quast C (2013). The SILVA ribosomal RNA gene database project: improved data processing and web-based tools. Nucleic Acids Res..

[47] Million M (2016). Increased Gut Redox and Depletion of Anaerobic and Methanogenic Prokaryotes in Severe Acute Malnutrition. Sci. Rep..

[48] Angelakis E (2016). Gut microbiome and dietary patterns in different Saudi populations and monkeys. Sci. Rep..

[49] Dhariwal A (2017). MicrobiomeAnalyst: a web-based tool for comprehensive statistical, visual and meta-analysis of microbiome data. Nucleic Acids Res..

[50] Huttenhower C (2012). Structure, function and diversity of the healthy human microbiome. Nature.

[51] Segata N (2011). Metagenomic biomarker discovery and explanation. Genome Biol..

