# Supplementary material for: Metagenomic and culturomic analysis of gut microbiota dysbiosis during Clostridium difficile infection
Source: Sci Rep. 2019 Sep 5;9:12807. doi: 10.1038/s41598-019-49189-8 (PMC6728329; doi:10.1038/s41598-019-49189-8)
Supplement: Supplementary file 1 — Supplementary data [file 41598_2019_49189_MOESM1_ESM.pdf]

Metagenomic and culturomic analysis of gut microbiota dysbiosis during *Clostridium difficile* infection.

Sophie Amrane[1], Marie Hocquart[1], Pamela Afouda[1], Edmond Kuete[1], Thi-Phuong-Thao Pham[1], Niokhor Dione[1], Issa Isaac Ngom[1], Camille Valles[1], Dipankar Bachar[1], Didier Raoult[1] and Jean Christophe Lagier[1]*.

[1]Aix Marseille Univ, IRD, MEPHI, IHU-Méditerranée Infection, Marseille, France

*To whom the correspondence should be addressed: jclagier@yahoo.fr

MEPHI, Aix Marseille Université, IRD, MEPHI

IHU - Méditerranée Infection

19-21 Boulevard Jean Moulin

13005 Marseille

Phone number: +33 4 13 73 24 01

Fax number: +33 4 13 73 24 02

SUPPLEMENTARY DATA:

Table S1: Six culture conditions selected for culturomic protocol.

**Culture conditions for culturomic protocol:**
Preincubation in aerobic blood culture bottle with 5 mL rumen fluid and then 5% sheep blood agar under aerobic conditions at 37°C
Preincubation in anaerobic blood culture bottle with 5mL rumen fluid and then 5% sheep blood agar under anaerobic conditions at 37°C
Preincubation in aerobic blood culture bottle with 5 ml sheep blood and then 5% sheep blood agar under aerobic conditions at 37°C
Preincubation in anaerobic blood culture bottle with 5 ml sheep blood and then 5% sheep blood agar under anaerobic conditions at 37°C
Preincubation in aerobic blood culture bottle with 5 ml rumen fluid and sheep blood and then 5% sheep blood agar under aerobic conditions at 37°C
Preincubation in anaerobic blood culture bottle with 5 ml rumen fluid and sheep blood and then 5% sheep blood agar under anaerobic conditions at 37°C

Table S2: Clinical characteristics of CDI patients analyzed by culturomics.

Patient	Sex	Age	Episode number	Severe colitis	Episode treatment	GenXpert	027 genotype	078 genotype	Immunosuppression	Antibiotic before first episode	Death caused by *C. difficile*
1	H	88	NR	NR	NA	B toxin + binary toxin -	Negative	Negative	NA	NA	NA
2	F	90	1st	No	Metronidazole	B toxin + binary toxin -	Negative	Positive	No	Piperacilline Tazobactam	NA
3	H	72	2nd	Yes (Renal failure)	FMT	B toxin + binary toxin +	Positive	Negative	No	Amoxicillin Clavulanic acid	Yes
4	H	58	5th	Yes (high leucocyte count, renal failure, pancolitis)	FMT	B toxin + binary toxin -	Negative	Negative	Yes (renal graft)	Cefepim	No
5	F	75	3rd	Non	Vancomycine Metronidazole	B toxin + binary toxin -	Negative	Negative	Yes (renal graft and low grade lymphoma)	Piperacilline Tazobactam	No
6	F	30	3rd	Non	FMT	B toxin + binary toxin -	Negative	Negative	No	Amoxicillin Clavulanic acid	No
7	H	79	2nd	Yes (high leucocyte count, renal failure, septic shock)	Fidaxomycine	B toxin + binary toxin -	Negative	Negative	No	Ciprofloxacin	No
8	F	92	1st	No	Vancomycine Metronidazole	B toxin + binary toxin -	Negative	Negative	No	Ceftriaxone	No
9	F	81	NA	NA	NA	B toxin + binary toxin -	Negative	Negative	Yes (pancreas cancer)	NA	No
10	F	41	2nd	No	FMT	B toxin + binary toxin -	Negative	Negative	No	Amoxicillin	No
11	F	73	2nd	No	FMT	B toxin + binary toxin -	Negative	Negative	No	Ceftriaxone Ciprofloxacin	No

Table S3: Bacteria detected in culturomics study. For each group (CDI and Controls), number of samples where each bacterium was detected. (Ctrl: Controls)

**Species**	**Origin**	**Phyla**	**All the controls n=8**	**All the CDI n=11**
Acidaminococcus intestini	H(GUT)	*Firmicutes*	2	1
Acinetobacter pittii	H	*Proteobacteria*	0	1
Actinomyces europaeus	H	*Actinobacteria*	2	0
Actinomyces mediterraneense	Newspe	*Actinobacteria*	1	0
Actinomyces neuii	H	*Actinobacteria*	0	1
Adlercreutzia equolifaciens	H(GUT)	*Actinobacteria*	3	0
Aeromonas hydrophila	H(GUT)	*Proteobacteria*	2	0
Alistipes caccae	Newspe	*Bacteroidetes*	1	0
Alistipes finegoldii	H(GUT)	*Bacteroidetes*	7	1
Alistipes indistinctus	H(GUT)	*Bacteroidetes*	6	0
Alistipes jeddahensis	Newspe	*Bacteroidetes*	5	2
Alistipes massiliensis	NH	*Bacteroidetes*	1	0
Alistipes obesi	Newspe	*Bacteroidetes*	8	1
Alistipes obesihominis	Newspe	*Bacteroidetes*	2	0
Alistipes onderdonkii	H(GUT)	*Bacteroidetes*	4	0
Alistipes putredinis	H(GUT)	*Bacteroidetes*	1	0
Alistipes senegalensis	Newspe	*Bacteroidetes*	5	0
Alistipes shahii	H(GUT)	*Bacteroidetes*	7	0
Alistipes timonensis	Newspe	*Bacteroidetes*	1	0
Allisonella histaminiformans	NH	*Firmicutes*	4	0
Anaerocella delicata	NH	*Bacteroidetes*	0	1
Anaerococcus murdochii	H	*Firmicutes*	1	0
Anaerococcus octavius	H	*Firmicutes*	1	0
Anaerococcus vaginalis	H(GUT)	*Firmicutes*	5	1
Anaerofustis massiliensis	Newspe	*Firmicutes*	1	0
Anaeroglobus geminatus	H	*Firmicutes*	1	2
Anaerosalibacter bizertensis	NH	*Firmicutes*	1	0
Anaerostipes caccae	H(GUT)	*Firmicutes*	0	1
Anaerotruncus colihominis	H(GUT)	*Firmicutes*	2	0
Anaerotruncus massiliensis	Newspe	*Firmicutes*	1	2
Anaerotruncus rubiinfantis	Newspe	*Firmicutes*	5	2
Arabia massiliensis	Newspe	*Actinobacteria*	1	0
Arcanobacterium urinimassiliense	H	*Actinobacteria*	2	0
Atopobium minutum	H(GUT)	*Actinobacteria*	2	0
Atopobium parvulum	H(GUT)	*Actinobacteria*	0	1
Bacillus amyloliquefaciens	H	*Firmicutes*	1	0
Bacillus anthracis	H(GUT)	*Firmicutes*	0	1
Bacillus cereus	H(GUT)	*Firmicutes*	3	2
Bacillus circulans	H(GUT)	*Firmicutes*	1	3
Bacillus clausii	H(GUT)	*Firmicutes*	1	0
Bacillus cytotoxicus	NH	*Firmicutes*	0	1

Bacillus farraginis	NH	*Firmicutes*	1	0
Bacillus licheniformis	H(GUT)	*Firmicutes*	1	4
Bacillus megaterium	H(GUT)	*Firmicutes*	0	1
Bacillus pumilus	H(GUT)	*Firmicutes*	1	4
Bacillus siralis	NH	*Firmicutes*	2	0
Bacillus sonorensis	H(GUT)	*Firmicutes*	0	1
Bacillus subtilis	H(GUT)	*Firmicutes*	2	2
Bacillus thermoamylovorans	H(GUT)	*Firmicutes*	1	0
Bacillus thioparans	NH	*Firmicutes*	1	0
Bacillus thuringiensis	H(GUT)	*Firmicutes*	1	0
Bacillus valismortis	NH	*Firmicutes*	1	3
Bacteroides bouchedurhonense	Newspe	*Bacteroidetes*	6	0
Bacteroides caccae	H(GUT)	*Bacteroidetes*	3	0
Bacteroides cellulosilyticus	H(GUT)	*Bacteroidetes*	3	0
Bacteroides congonense	Newspe	*Bacteroidetes*	1	0
Bacteroides eggerthii	H(GUT)	*Bacteroidetes*	2	0
Bacteroides fragilis	H(GUT)	*Bacteroidetes*	3	0
Bacteroides intestinalis	H(GUT)	*Bacteroidetes*	3	1
Bacteroides massiliensis	H	*Bacteroidetes*	3	0
Bacteroides nordii	H(GUT)	*Bacteroidetes*	2	0
Bacteroides oleiciplenus	H(GUT)	*Bacteroidetes*	1	0
Bacteroides ovatus	H(GUT)	*Bacteroidetes*	8	0
Bacteroides salyersiae	H(GUT)	*Bacteroidetes*	2	0
Bacteroides stercoris	H(GUT)	*Bacteroidetes*	3	0
Bacteroides thetaiotaomicron	H(GUT)	*Bacteroidetes*	6	0
Bacteroides timonense	Newspe	*Bacteroidetes*	1	0
Bacteroides uniformis	H(GUT)	*Bacteroidetes*	8	1
Bacteroides vulgatus	H(GUT)	*Bacteroidetes*	8	0
Bariatricus massiliensis	Newspe	*Firmicutes*	0	1
Barnesiella intestinihominis	H(GUT)	*Bacteroidetes*	6	0
Beduinella massiliensis	Newspe	*Firmicutes*	5	1
Bifidobacterium adolescentis	H(GUT)	*Actinobacteria*	6	0
Bifidobacterium animalis	H(GUT)	*Actinobacteria*	3	0
Bifidobacterium bifidum	H(GUT)	*Actinobacteria*	5	0
Bifidobacterium catenulatum	H(GUT)	*Actinobacteria*	3	0
Bifidobacterium longum	H(GUT)	*Actinobacteria*	6	0
Bifidobacterium pseudocatenulatum	H(GUT)	*Actinobacteria*	4	0
Bifidobacterium ruminantium	NH	*Actinobacteria*	1	0
Bilophila wadsworthia	H(GUT)	*Proteobacteria*	5	0
Bittarella massiliensis	Newspe	*Firmicutes*	2	0
Blautia coccoides	H(GUT)	*Firmicutes*	2	6
Blautia hydrogenotrophica	H(GUT)	*Firmicutes*	4	3
Blautia ihumii	Newspe	*Firmicutes*	1	0
Blautia marasmi	Newspe	*Firmicutes*	0	1
Blautia mediterraneensis	Newspe	*Firmicutes*	1	0
Blautia phocaeensis	Newspe	*Firmicutes*	4	0

Blautia timonensis	Newspe	*Firmicutes*	1	0
Butyricimonas phoceensis	Newspe	*Bacteroidetes*	6	0
Butyricimonas virosa	H	*Bacteroidetes*	5	0
Caecumella massiliensis	Newspe	*Firmicutes*	1	0
Campylobacter concisus	H(GUT)	*Proteobacteria*	1	0
Campylobacter ureolyticus	H(GUT)	*Proteobacteria*	1	0
Candida albicans	Fungi	*Fungi*	0	3
Candida glabrata	Fungi	*Fungi*	0	1
Candida kefyr	Fungi	*Fungi*	0	1
Candida tropicalis	Fungi	*Fungi*	0	1
Casaltella massiliensis	H	*Firmicutes*	1	0
Catabacter hongkongensis	H(GUT)	*Firmicutes*	4	0
Christensenella massiliensis	Newspe	*Firmicutes*	1	0
Christensenella minuta	H(GUT)	*Firmicutes*	1	0
Chryseobacterium gleum	H	*Bacteroidetes*	0	1
Citrobacter amalonaticus	H(GUT)	*Proteobacteria*	0	1
Citrobacter freundii	H(GUT)	*Proteobacteria*	0	2
Citrobacter koseri	H(GUT)	*Proteobacteria*	0	2
Clostridium aldenense	H(GUT)	*Firmicutes*	4	3
Clostridium aminovalericum	H(GUT)	*Firmicutes*	2	0
Clostridium beduini	Newspe	*Firmicutes*	2	0
Clostridium bifermentans	H(GUT)	*Firmicutes*	5	2
Clostridium bolteae	H(GUT)	*Firmicutes*	6	3
Clostridium bouchedurhonense	Newspe	*Firmicutes*	4	0
Clostridium butyricum	H(GUT)	*Firmicutes*	0	4
Clostridium citroniae	H(GUT)	*Firmicutes*	3	0
Clostridium clostridioforme	H(GUT)	*Firmicutes*	7	6
Clostridium culturomicsense	Newspe	*Firmicutes*	1	2
Clostridium difficile	H(GUT)	*Firmicutes*	0	8
Clostridium disporicum	H(GUT)	*Firmicutes*	1	0
Clostridium glycolicum	Newspe	*Firmicutes*	0	3
Clostridium glycyrrhizinilyticum	H(GUT)	*Firmicutes*	2	0
Clostridium hathewayi	H(GUT)	*Firmicutes*	6	4
Clostridium innocuum	H(GUT)	*Firmicutes*	5	7
Clostridium jeddahmassiliense	Newspe	*Firmicutes*	0	2
Clostridium jeddahtimonense	Newspe	*Firmicutes*	0	1
Clostridium lavalense	H(GUT)	*Firmicutes*	7	1
Clostridium limosum	H	*Firmicutes*	0	1
Clostridium nondifficile	My newspe	*Firmicutes*	0	1
Clostridium paraperfringens	Newspe	*Firmicutes*	0	3
Clostridium paraputrificum	H(GUT)	*Firmicutes*	4	8
Clostridium perfringens	H(GUT)	*Firmicutes*	4	6
Clostridium phoceense	Newspe	*Firmicutes*	2	1
Clostridium putrefaciens	H(GUT)	*Firmicutes*	1	0
Clostridium ramosum	H(GUT)	*Firmicutes*	3	5
Clostridium saccharogumia	H(GUT)	*Firmicutes*	1	0

Clostridium sartagoforme	NH	*Firmicutes*	0	1
Clostridium sartagoforme	NH	*Firmicutes*	1	0
Clostridium saudii	Newspe	*Firmicutes*	2	0
Clostridium sauditimonense	Newspe	*Firmicutes*	0	2
Clostridium scindens	H(GUT)	*Firmicutes*	4	1
Clostridium sordellii	H(GUT)	*Firmicutes*	3	4
Clostridium sporogenes	H(GUT)	*Firmicutes*	4	2
Clostridium sulfidigenes	NH	*Firmicutes*	1	0
Clostridium symbiosum	H(GUT)	*Firmicutes*	4	4
Clostridium tertium	H(GUT)	*Firmicutes*	4	9
Clostridium tyrobutyricum	H(GUT)	*Firmicutes*	1	2
Collinsella aerofaciens	H(GUT)	*Actinobacteria*	5	0
Collinsella massiliensis	Newspe	*Actinobacteria*	2	0
Collinsella tanakaei	H(GUT)	*Actinobacteria*	1	0
Coprobacillus cateniformis	H(GUT)	*Firmicutes*	2	2
Coprobacillus massiliensis	My newspe	*Firmicutes*	0	1
Corynebacterium amycolatum	H	*Actinobacteria*	0	1
Corynebacterium tuberculostearicum	H	*Actinobacteria*	0	2
Dakarella massiliensis	Newspe	*Proteobacteria*	3	0
Desulfovibrio piger	H(GUT)	*Proteobacteria*	1	0
Dialister invisus	H	*Firmicutes*	3	1
Dialister pneumosintes	H	*Firmicutes*	3	0
Dialister succinatiphilus	H(GUT)	*Firmicutes*	2	0
Dielma fastidiosa	Newspe	*Firmicutes*	3	0
Dorea massiliensis	Newspe	*Firmicutes*	1	0
Dorea timonensis	Newspe	*Firmicutes*	2	0
Drancourtella massiliensis	Newspe	*Firmicutes*	1	3
Duodena massiliensis	Newspe	*Proteobacteria*	2	0
Eggerthella lenta	H(GUT)	*Actinobacteria*	7	6
Eggerthella timonensis	Newspe	*Actinobacteria*	1	0
Eisenbergiella massiliensis	Newspe	*Firmicutes*	4	2
Enterobacter cloacae	H(GUT)	*Proteobacteria*	1	1
Enterococcus avium	H(GUT)	*Firmicutes*	3	6
Enterococcus canintestini	H	*Firmicutes*	0	1
Enterococcus casseliflavus	H(GUT)	*Firmicutes*	1	6
Enterococcus devriesei	NH	*Firmicutes*	1	1
Enterococcus dispar	H(GUT)	*Firmicutes*	1	0
Enterococcus durans	H(GUT)	*Firmicutes*	3	1
Enterococcus faecalis	H(GUT)	*Firmicutes*	4	10
Enterococcus faecium	H(GUT)	*Firmicutes*	7	11
Enterococcus gallinarum	H(GUT)	*Firmicutes*	3	5
Enterococcus hirae	H(GUT)	*Firmicutes*	2	2
Enterococcus malodoratum	H	*Firmicutes*	0	2
Enterococcus massiliensis	Newspe	*Firmicutes*	0	1
Enterococcus pseudoavium	H	*Firmicutes*	0	1
Enterococcus thailandicus	NH	*Firmicutes*	0	1

Escherichia coli	H(GUT)	*Proteobacteria*	7	7
Escherichia hermannii	H(GUT)	*Proteobacteria*	1	0
Eubacterium contortum	H	*Firmicutes*	0	1
Eubacterium cylindroides	H(GUT)	*Firmicutes*	1	0
Eubacterium limosum	H(GUT)	*Firmicutes*	3	0
Eubacterium massiliense	Newspe	*Firmicutes*	5	0
Eubacterium tenue	H(GUT)	*Firmicutes*	2	1
Finegoldia magna	H(GUT)	*Firmicutes*	1	1
Flavonifractor plautii	H(GUT)	*Firmicutes*	7	7
Fusobacterium naviforme	H	*Fusobacteria*	0	1
Fusobacterium nucleatum	H(GUT)	*Fusobacteria*	0	1
Gabonia massiliensis	Newspe	*Bacteroidetes*	2	0
Gabonibacter massiliensis	Newspe	*Bacteroidetes*	1	0
Gabonibacter timonensis	Newspe	*Bacteroidetes*	1	0
Gordonibacter pamelaeae	H(GUT)	*Actinobacteria*	5	1
Gordonibacter urolithinfaciens	H(GUT)	*Actinobacteria*	2	0
Granulicatella adiacens	H(GUT)	*Firmicutes*	0	1
Granulicatella elegans	H	*Firmicutes*	0	1
Guyana massiliensis	Newspe	*Unclassified bacteria*	1	0
Haemophilus haemolyticus	H	*Proteobacteria*	1	0
Hafnia alvei	H(GUT)	*Proteobacteria*	0	2
Holdemanella biformis	NH	*Firmicutes*	4	0
Holdemania filiformis	H(GUT)	*Firmicutes*	2	0
Holdemania massiliensis	Newspe	*Firmicutes*	6	0
Holdemania timonensis	Newspe	*Firmicutes*	3	2
Ihubacter massiliensis	Newspe	*Firmicutes*	1	0
Ihuella massiliensis	Newspe	*Firmicutes*	4	0
Intestinibacter bartlettii	NH	*Firmicutes*	3	0
Intestinimonas butyriciproducens	NH	*Firmicutes*	3	1
Intestinimonas massiliensis	Newspe	*Firmicutes*	3	0
Intestinimonas phoceensis	Newspe	*Firmicutes*	1	0
Jeddahella massiliensis	Newspe	*Unclassified bacteria*	1	0
Klebsiella oxytoca	H(GUT)	*Proteobacteria*	1	2
Klebsiella pneumoniae	H(GUT)	*Proteobacteria*	1	5
Kluyvera ascorbata	H(GUT)	*Proteobacteria*	2	0
Kocuria rhizophila	H	*Actinobacteria*	1	0
Lachnoclostridium edouardi	Newspe	*Firmicutes*	1	0
Lachnoclostridium timonense	Newspe	*Firmicutes*	0	1
Lactobacillus agilis	NH	*Firmicutes*	0	1
Lactobacillus brevis	H(GUT)	*Firmicutes*	0	1
Lactobacillus casei	H(GUT)	*Firmicutes*	0	3
Lactobacillus fermentum	H(GUT)	*Firmicutes*	0	3
Lactobacillus gasseri	H(GUT)	*Firmicutes*	0	1
Lactobacillus harbinensis	NH	*Firmicutes*	0	1
Lactobacillus johnsonii	H(GUT)	*Firmicutes*	0	1
Lactobacillus minutum	Newspe	*Firmicutes*	1	0

Lactobacillus mucosae	H(GUT)	*Firmicutes*	1	0
Lactobacillus otakiensis	NH	*Firmicutes*	0	1
Lactobacillus parabuchneri	H	*Firmicutes*	0	2
Lactobacillus paracasei	H(GUT)	*Firmicutes*	0	5
Lactobacillus parafarraginis	NH	*Firmicutes*	0	2
Lactobacillus pentosus	NH	*Firmicutes*	0	2
Lactobacillus plantarum	H(GUT)	*Firmicutes*	0	3
Lactobacillus raoultii	Newspe	*Firmicutes*	0	1
Lactobacillus rapi	NH	*Firmicutes*	0	1
Lactobacillus reuteri	H(GUT)	*Firmicutes*	2	3
Lactobacillus rhamnosus	H(GUT)	*Firmicutes*	1	4
Lactobacillus ruminis	H(GUT)	*Firmicutes*	3	0
Lactobacillus salivarius	H(GUT)	*Firmicutes*	0	4
Lactobacillus timonensis	Newspe	*Firmicutes*	1	0
Lactobacillus vaginalis	H(GUT)	*Firmicutes*	1	0
Lactococcus lactis	H(GUT)	*Firmicutes*	2	0
Lactonifactor massiliensis	Newspe	*Firmicutes*	1	0
Leuconostoc lactis	H(GUT)	*Firmicutes*	0	1
Lysinibacillus boronitolerans	NH	*Firmicutes*	1	0
Lysinibacillus fusiformis	H(GUT)	*Firmicutes*	1	3
Macrococcus caseolyticus	H	*Firmicutes*	0	1
Marseillobacter massiliensis	Newspe	*Firmicutes*	2	0
Marseillococcus timonensis	Newspe	*Firmicutes*	1	0
Massilicoli timonensis	Newspe	*NR*	0	1
Massilimaliae timonensis	Newspe	*Firmicutes*	2	0
Massilioculturomica massiliensis	Newspe	*Firmicutes*	1	0
Massiliomaliae massiliensis	Newspe	*Firmicutes*	3	0
Massiliomicrobiota timonensis	Newspe	*Firmicutes*	1	1
Massilioprevotella massiliensis	Newspe	*Bacteroidetes*	3	0
Mediannikovella massiliensis	Newspe	*Firmicutes*	1	0
Megasphaera indica	H(GUT)	*Firmicutes*	3	0
Megasphaera micronuciformis	H (gut)	*Firmicutes*	0	1
Micrococcus luteus	H(GUT)	*Actinobacteria*	1	4
Micromassilia timonensis	Newspe	*Firmicutes*	1	0
Mitsuokella jalaludinii	NH	*Firmicutes*	1	0
Mobilibacterium timonense	Newspe	*Firmicutes*	1	0
Mobiluncus curtisii	H	*Actinobacteria*	1	0
Mogibacterium timidum	H(GUT)	*Firmicutes*	4	0
Mogibacterium vescum	H	*Firmicutes*	1	0
Morganella morganii	H(GUT)	*Proteobacteria*	0	3
Murdochiella massiliensis	Newspe	*Firmicutes*	1	0
Negativibacillus massiliensis	Newspe	*Firmicutes*	1	0
Negativicoccus massiliensis	Newspe	*Firmicutes*	1	0
Neisseria flavescens	H	*Proteobacteria*	0	1
Neisseria perflava	H	*Proteobacteria*	0	1
Neisseria subflava	H (gut)	*Proteobacteria*	0	1

Neochristensenella massiliensis	Newspe	*Firmicutes*	1	0
Odoribacter splanchnicus	H(GUT)	*Bacteroidetes*	3	1
Olegusella massiliensis	Newspe	*Actinobacteria*	1	0
Olsenella phoceensis	Newspe	*Actinobacteria*	3	0
Olsenella timonensis	Newspe	*Actinobacteria*	5	0
Olsenella uli	H	*Actinobacteria*	6	1
Oscillibacter massiliensis	Newspe	*Firmicutes*	8	0
Oscillibacter timonensis	Newspe	*Firmicutes*	1	0
Paenibacillus lautus	H(GUT)	*Firmicutes*	0	1
Parabacteroides bouchesdurhonensis	Newspe	*Bacteroidetes*	1	0
Parabacteroides distasonis	H(GUT)	*Bacteroidetes*	6	1
Parabacteroides johnsonii	H(GUT)	*Bacteroidetes*	1	0
Parabacteroides massiliense	Newspe	*Bacteroidetes*	1	0
Parabacteroides merdae	H(GUT)	*Bacteroidetes*	7	0
Parabacteroides provencensis	Newspe	*Bacteroidetes*	1	0
Parasutterella excrementihominis	H(GUT)	*Proteobacteria*	5	1
Pediococcus acidilactici	H (gut)	*Firmicutes*	0	1
Pediococcus pentosaceus	H(GUT)	*Firmicutes*	0	2
Peptoniphilus asaccharolyticus	H(GUT)	*Firmicutes*	1	0
Peptoniphilus gorbachii	H(GUT)	*Firmicutes*	2	0
Peptoniphilus grossensis	Newspe	*Firmicutes*	4	0
Peptoniphilus harei	H	*Firmicutes*	1	0
Peptoniphilus obesi	Newspe	*Firmicutes*	1	0
Peptoniphilus obesihominis	Newspe	*Firmicutes*	1	0
Peptostreptococcus anaerobius	H(GUT)	*Firmicutes*	1	0
Phascolarctobacterium faecium	H(GUT)	*Firmicutes*	4	0
Phocea massiliensis	Newspe	*Firmicutes*	3	0
Polynesia massiliensis	Newspe	*Firmicutes*	5	1
Prevotella caldimerdae	Newspe	*Bacteroidetes*	1	0
Prevotella copri	H(GUT)	*Bacteroidetes*	2	0
Prevotella corporis	H	*Bacteroidetes*	1	0
Prevotella provencensis	H	*Bacteroidetes*	1	0
Prevotella salivae	H	*Bacteroidetes*	2	0
Propionibacterium acnes	H(GUT)	*Actinobacteria*	4	4
Propionibacterium avidum	H(GUT)	*Actinobacteria*	0	1
Propionimicrobium lymphophilum	H	*Actinobacteria*	1	0
Proteus hauseri	H	*Proteobacteria*	0	1
Proteus mirabilis	H(GUT)	*Proteobacteria*	0	2
Proteus penneri	H (gut)	*Proteobacteria*	0	1
Proteus vulgaris	H(GUT)	*Proteobacteria*	0	3
Provencella massiliensis	Newspe	*Firmicutes*	6	0
Providencia rettgeri	H (gut)	*Proteobacteria*	0	1
Pseudochrobactrum assaccharolyticum	H	*Proteobacteria*	1	0
Pseudoflavonifractor capillosus	H(GUT)	*Firmicutes*	1	0
Pseudomonas aeruginosa	H(GUT)	*Proteobacteria*	0	2
Pseudoramibacter alactolyticus	H	*Firmicutes*	3	0

Pyramidobacter piscolens	H(GUT)	*Synergistetes*	4	0
Raoultibacter massiliensis	Newspe	*Actinobacteria*	3	0
Raoultibacter timonensis	Newspe	*Actinobacteria*	1	0
Robinsoniella peoriensis	H(GUT)	*Firmicutes*	0	1
Romboutsia lituseburensis	H(GUT)	*Firmicutes*	1	0
Ruminiclostridium massiliense	Newspe	*Firmicutes*	5	0
Ruminococcus gen nov P3876P	Newspe	*Firmicutes*	1	0
Ruminococcus gnavus	H(GUT)	*Firmicutes*	0	3
Ruminococcus phoceensis	Newspe	*Firmicutes*	2	0
Ruthenibacterium lactatiformans	H(GUT)	*Firmicutes*	4	0
Salmonella sp.	H(GUT)	*Proteobacteria*	0	1
Sanguibacteroides massiliense	Newspe	*Bacteroidetes*	1	0
Senegalia massiliensis	Newspe	*Firmicutes*	1	0
Senegalimassilia anaerobia	Newspe	*Actinobacteria*	3	0
Senegalobactrium massiliense	Newspe	*Firmicutes*	1	0
Serratia fonticola	H(GUT)	*Proteobacteria*	2	0
Slackia exigua	H	*Actinobacteria*	1	0
Slackia isoflavoniconvertens	H(GUT)	*Actinobacteria*	1	0
Staphylococcus capitis	H(GUT)	*Firmicutes*	0	2
Staphylococcus epidermidis	H(GUT)	*Firmicutes*	3	3
Staphylococcus haemolyticus	H(GUT)	*Firmicutes*	0	2
Staphylococcus hominis	H(GUT)	*Firmicutes*	1	1
Staphylococcus kloosi	H	*Firmicutes*	1	0
Staphylococcus xylosus	H(GUT)	*Firmicutes*	1	1
Streptococcus anginosus	H(GUT)	*Firmicutes*	2	0
Streptococcus australis	NH	*Firmicutes*	0	1
Streptococcus constellatus	H(GUT)	*Firmicutes*	2	0
Streptococcus gallolyticus	H(GUT)	*Firmicutes*	1	0
Streptococcus infantarius	H(GUT)	*Firmicutes*	1	1
Streptococcus lutetiensis	H(GUT)	*Firmicutes*	1	0
Streptococcus mitis	H(GUT)	*Firmicutes*	2	1
Streptococcus oralis	H(GUT)	*Firmicutes*	2	0
Streptococcus salivarius	H(GUT)	*Firmicutes*	2	1
Streptococcus sanguinis	H(GUT)	*Firmicutes*	1	1
Sutterella massiliensis	Newspe	*Proteobacteria*	1	0
Sutterella wadsworthensis	H(GUT)	*Proteobacteria*	6	1
Terrisporobacter mayombei	Newspe	*Firmicutes*	1	0
Tidjanibacter massiliensis	Newspe	*Bacteroidetes*	1	0
Togobacter massiliensis	Newspe	*Firmicutes*	0	1
Ureibacillus thermosphaericus	H(GUT)	*Firmicutes*	1	0
Urmitella massiliensis	Newspe	*Firmicutes*	0	1
Vagococcus fluvialis	H	*Firmicutes*	0	1
Veillonella atypica	H(GUT)	*Firmicutes*	1	1
Veillonella parvula	H (gut)	*Firmicutes*	0	2
Virgibacillus dakarensis	Newspe	*Firmicutes*	1	0
Virgibacillus proomii	NH	*Firmicutes*	1	0

Table S4: OTUs repartition between CDI and control group.

	OTUs detected only in CDI group	OTUs detected in both groups	OTUs detected only in control group
OTUs	541	728	1049
OTUs assigned to a known species	220	232	290

Figure S1: LDA score with metagenomic data from CDI and control group.

Figure S2: Bacteria associated to healthy or CDI microbiota, found by culturomics and metagenomics.

Figure S3: Cytoscape representation of bacteria detected in culturomic and metagenomic analysis up to the genera level. A) CDI group, B) Control group.

Figure S1:

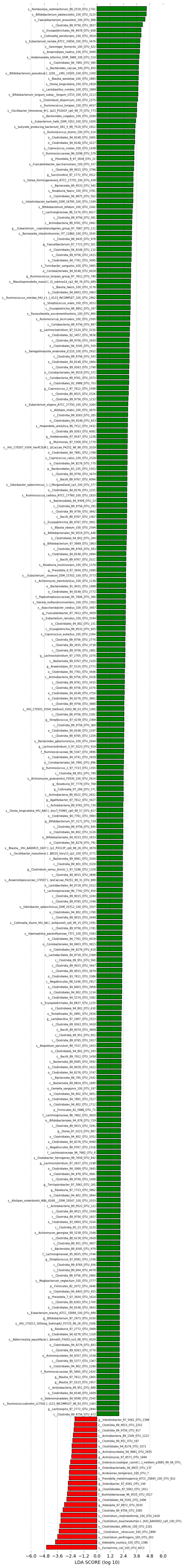

Figure S2:

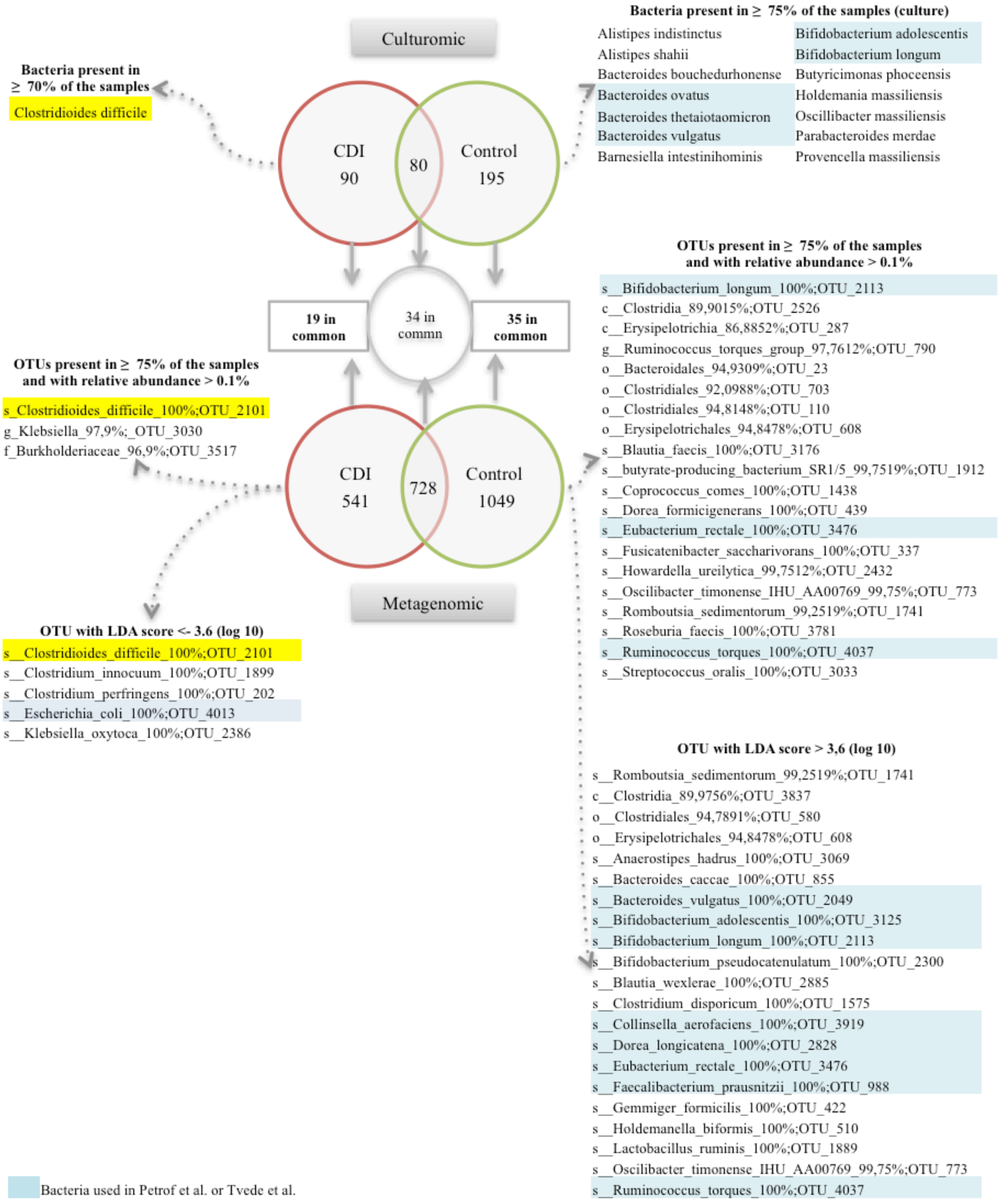

Culturomic
CDI 90
80
Control 195
19 in common
34 in commn
35 in common
CDI 541
728
Control 1049
Metagenomic
Bacteria present in ≥ 70% of the samples
Clostridioides difficile
Bacteria present in ≥ 75% of the samples (culture)
Alistipes indistinctus
Alistipes shahii
Bacteroides bouchedurhonense
Bacteroides ovatus
Bacteroides thetaiotaomicron
Bacteroides vulgatus
Barnesiella intestinihominis
Bifidobacterium adolescentis
Bifidobacterium longum
Butyricimonas phoceensis
Holdemania massiliensis
Oscillibacter massiliensis
Parabacteroides merdae
Provencella massiliensis
OTUs present in ≥ 75% of the samples and with relative abundance > 0.1%
s_Clostridioides_difficile_100%;OTU_2101
g_Klebsiella_97,9%;_OTU_3030
f_Burkholderiaceae_96,9%;OTU_3517
OTUs present in ≥ 75% of the samples and with relative abundance > 0.1%
s__Bifidobacterium_longum_100%;OTU_2113
c__Clostridia_89,9015%;OTU_2526
c__Erysipelotrichia_86,8852%;OTU_287
g__Ruminococcus_torques_group_97,7612%;OTU_790
o__Bacteroidales_94,9309%;OTU_23
o__Clostridiales_92,0988%;OTU_703
o__Clostridiales_94,8148%;OTU_110
o__Erysipelotrichales_94,8478%;OTU_608
s__Blautia_faecis_100%;OTU_3176
s__butyrate-producing_bacterium_SR1/5_99,7519%;OTU_1912
s__Coprococcus_comes_100%;OTU_1438
s__Dorea_formicigenerans_100%;OTU_439
s__Eubacterium_rectale_100%;OTU_3476
s__Fusicatenibacter_saccharivorans_100%;OTU_337
s__Howardella_ureilytica_99,7512%;OTU_2432
s__Oscilibacter_timonense_IHU_AA00769_99,75%;OTU_773
s__Romboutsia_sedimentorum_99,2519%;OTU_1741
s__Roseburia_faecis_100%;OTU_3781
s__Ruminococcus_torques_100%;OTU_4037
s__Streptococcus_oralis_100%;OTU_3033
OTU with LDA score <- 3.6 (log 10)
s__Clostridioides_difficile_100%;OTU_2101
s__Clostridium_innocuum_100%;OTU_1899
s__Clostridium_perfringens_100%;OTU_202
s__Escherichia_coli_100%;OTU_4013
s__Klebsiella_oxytoca_100%;OTU_2386
OTU with LDA score > 3,6 (log 10)
s__Romboutsia_sedimentorum_99,2519%;OTU_1741
c__Clostridia_89,9756%;OTU_3837
o__Clostridiales_94,7891%;OTU_580
o__Erysipelotrichales_94,8478%;OTU_608
s__Anaerostipes_hadrus_100%;OTU_3069
s__Bacteroides_caccae_100%;OTU_855
s__Bacteroides_vulgatus_100%;OTU_2049
s__Bifidobacterium_adolescentis_100%;OTU_3125
s__Bifidobacterium_longum_100%;OTU_2113
s__Bifidobacterium_pseudocatenulatum_100%;OTU_2300
s__Blautia_wexlerae_100%;OTU_2885
s__Clostridium_disporicum_100%;OTU_1575
s__Collinsella_aerofaciens_100%;OTU_3919
s__Dorea_longicatena_100%;OTU_2828
s__Eubacterium_rectale_100%;OTU_3476
s__Faecalibacterium_prausnitzii_100%;OTU_988
s__Gemmiger_formicilis_100%;OTU_422
s__Holdemanella_biformis_100%;OTU_510
s__Lactobacillus_ruminis_100%;OTU_1889
s__Oscilibacter_timonense_IHU_AA00769_99,75%;OTU_773
s__Ruminococcus_torques_100%;OTU_4037
Bacteria used in Petrof et al. or Tvede et al.

Figure S3:

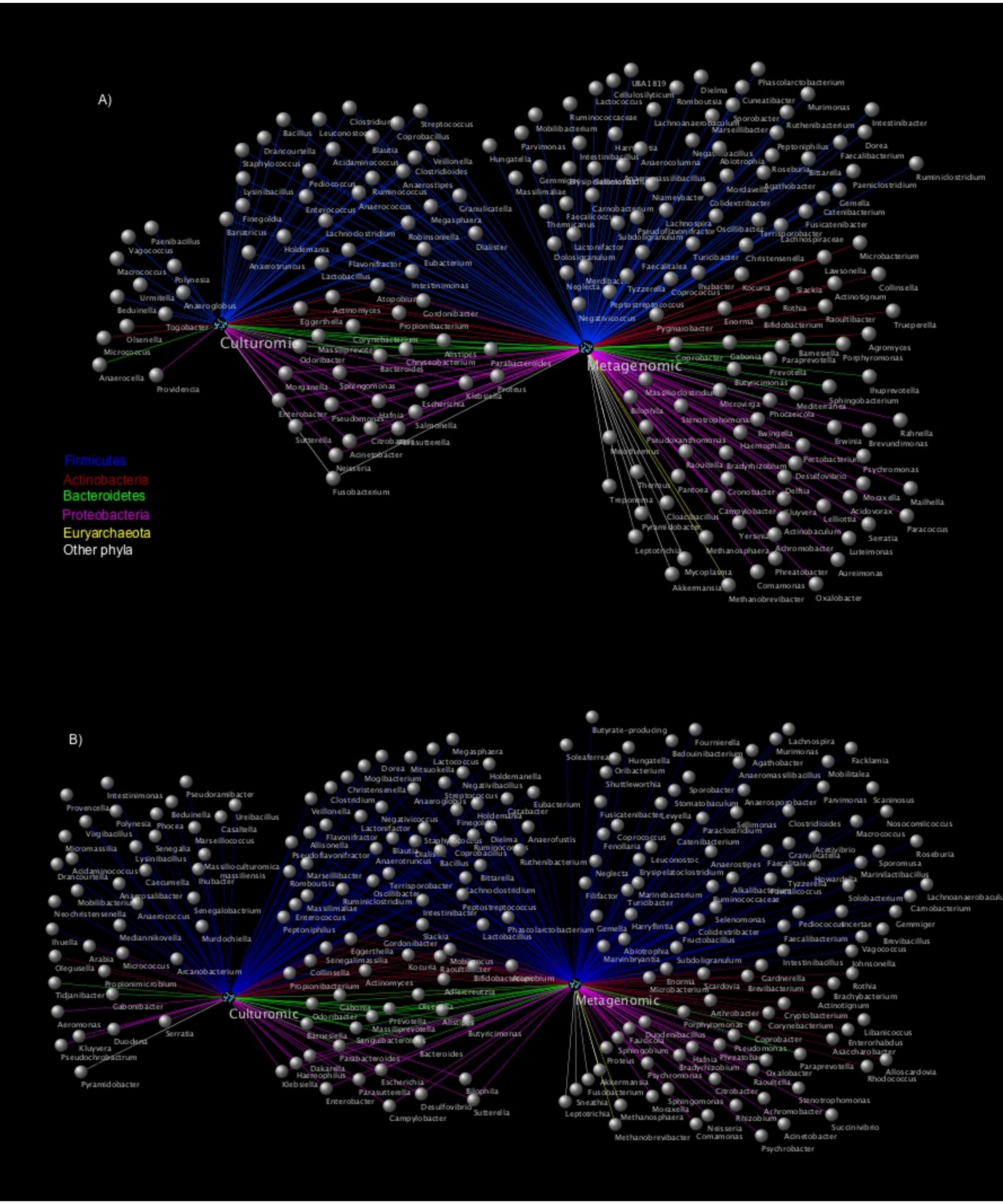
A)
Culturomic
Metagenomic
Firmicutes
Actinobacteria
Bacteroidetes
Proteobacteria
Euryarchaeota
Other phyla
B)
Culturomic
Metagenomic
